# Comparison of total oxidative state, total antioxidative state, serum paraoxonase activity, and thiol levels before and after 6 months of treatment in patients with multiple sclerosis who have been recently started on new therapy agents

**DOI:** 10.55730/1300-0144.5983

**Published:** 2025-03-10

**Authors:** Emel BAŞAR, Hasan BAYINDIR, Hatice Ferhan KÖMÜRCÜ, Ebru Bilge DİRİK, Ömer ANLAR, Özcan EREL

**Affiliations:** 1 Department of Neurology, Ankara Bilkent City Hospital, Ankara, Turkiye; 2 Department of Neurology, University of Health Sciences, Fatih Sultan Mehmet Training and Research Hospital, İstanbul, Turkiye; 3 Department of Neurology, Yüksek İhtisas University Medical School, Ankara, Turkiye; 4 Department of Clinical Biochemistry, Yıldırım Beyazıt University Medical School, Ankara, Turkiye

**Keywords:** Multiple sclerosis, oxidative stress, immunomodulatory therapy

## Abstract

**Background/aim:**

The objective of this study was to establish the oxidative stress determiners as a total oxidative state (TOS), total antioxidative state (TAS), paraoxonase (PON) activity, oxidative stress index (OSI), and thiol/disulfide levels before and after 6 months of immunomodulator therapy in patients who were diagnosed according to the revised McDonald Criteria.

**Materials and methods:**

Thirty-nine patients were included in our study who were diagnosed according to the revised McDonald Criteria, approved to participate in the study, and had been treated in the neurology inpatient clinic or presented in the neurology outpatient clinic of Ankara Yıldırım Beyazıt University Atatürk Training and Research Hospital. Blood samples were collected from patients before treatment and six months after treatment. The venous blood samples of the patients were evaluated for routine biochemical tests, TOS, TAS, PON activity, OSI, and thiol/disulfide levels.

**Results:**

The oxidative stress parameter levels of blood samples obtained before and after 6 months of the treatment were compared. The blood samples obtained after 6 months of therapy exhibited statistically significant results, including elevated levels of native thiol/total thiol (SH/SH+SS), PON, and TAS (p = 0.001). No significant relation was observed between the duration of disease, sex, used therapy agents, and oxidative stress parameters.

**Conclusion:**

In this study, we determined that TOS and OSI, which are indicators of oxidative stress, and TAS and PON, which exhibit antioxidative activity, can be employed to evaluate the therapy response. Additionally, we found that immunomodulator therapies can exhibit a protective effect, as evidenced by a reduction in oxidative stress indicators.

## 1. Introduction

Multiple sclerosis (MS), a chronic condition affecting the central nervous system, typically advances through a combination of autoimmune inflammation leading to demyelination and ongoing neurodegeneration, resulting in periods of exacerbation and/or gradual disability [1,2]. The condition is characterized by inflammation occurring at multiple sites, alongside the loss of myelin, proliferation of glial cells, and neuronal degeneration within the brain and spinal cord [3]. Although a genetic predisposition is recognized as a contributing factor to the development of MS, there is a substantial emphasis on environmental factors that are believed to trigger the pathological mechanisms that cause the disease [4]. MS is a disease that is more prevalent in adolescents and young adults, but it can be observed at any age. Typically, symptoms of multiple sclerosis appear between the ages of 20 and 40. The incidence in women is more than double that of men [5].

The main feature of MS lesions is demyelination with destruction of the myelin sheath while the axon is relatively preserved. It is known that axonal degeneration, which develops in addition to demyelination, is the main cause of neurological weakness. From this perspective, MS can be characterized as a condition involving inflammation, demyelination, and neurodegeneration within the nervous system [6]. The pathological features are characterized by blood-brain barrier (BBB) damage and inflammatory cell infiltration, oligodendrocyte damage and demyelination, astrocytosis, axon damage and limited remyelination [7].

Oxidative stress occurs due to the accumulation of reactive oxygen and nitrogen species in cellular processes, leading to cellular damage and resulting in necrosis or apoptosis [8]. The brain is particularly susceptible to oxidative stress, which plays a significant role in the pathophysiology of neurological disorders [9]. The overall oxidant level, indicated by Total Oxidant Status (TOS), serves as a rough measure of the cumulative oxidative stress within the organism. Predominantly found in plasma, these oxidant substances encompass reactive oxygen and nitrogen derivatives, along with compounds like homocysteine, myeloperoxidase, and lipoxygenases [10]. It is thought that measuring total antioxidant capacity provides more valuable information than measuring antioxidants individually [11]. During the disease process, cytokines such as TNF-α and IFN-γ increase the production of nitric oxide (NO). Nitric oxide reacts with superoxide to form peroxynitrite, which leads to damage of the blood-brain barrier (BBB) and axonal loss. Oxidative stress accelerates disease progression by impairing the repair of myelin [8].

The objective of this study was to establish the oxidative stress determiners as TOS, total antioxidative state (TAS), paraoxonase (PON) activity, oxidative stress index (OSI), and thiol/disulfide levels before and after 6 months of immunomodulator therapy in patients who were diagnosed according to the revised McDonald Criteria. Additionally, the study aimed to contribute to the development of new biomarkers by determining the relationship between multiple sclerosis patients and oxidative stress throughout the disease and in response to therapy.

## 2. Materials and methods

This prospective and cross-sectional study included 40 patients who had received treatment at the neurology inpatient clinic or visited the neurology outpatient clinic at Ankara Yıldırım Beyazıt University Atatürk Training and Research Hospital. A documented informed consent was provided by each participant. Confirmed diagnosis of multiple sclerosis in accordance with the revised McDonald Criteria [5]. One patient was excluded from the investigation due to his failure to complete the six-month treatment regimen. Five cc of the patients’ venous blood was taken in EDTA tubes twice: immediately before therapy began and 6 months later, following an eight-h fast. Following centrifugation at 4000 rpm for 10 min in the biochemistry laboratory using the Nüve brand NF500R devices, the sera were stored at −80°C until biochemical analysis. Following a 12-h period at +4 °C to thaw the samples, biochemical analysis was performed on the same day. Using Rel Assay commercial kits on a Roche Hitachi Cobas c501 automated analyzer, oxidative stress markers, such as TAS, TOS, PON, total thiol, native thiol, and disulfide levels in patient samples, were examined. The OSI was determined using the formula: OSI (arbitrary unit) = [TOS (μmol H2O2 Eqv/L)/(TAS) (mmol Trolox Eqv/L)] × 100. For statistical analysis, the mean ± standard deviation (mean ± SD), t-test, and Pearson correlation analysis were conducted using SPSS for Windows version 11.5. The significance threshold for statistical significance was set at 0.05.

## 3. Results

A total of 39 patients who fulfilled the necessary criteria were included in the study. The mean age of the patients is 39.26 ± 10.52. Out of the total cases, 66.7% (n = 26) were female and 33.3% (n = 13) were male. Teriflunomide was given to 43.6% of the patients, interferon beta-1b to 7.7%, glatiramer acetate to 2.6%, interferon beta-1a to 23.1%, fingolimod to 20.5%, and dimethyl fumarate to 2.6%. The mean length of disease is 4.03 ± 3.12 years, with a median of 4 years, and the range is from 1 year to 11 years.

Measurements of oxidative stress parameters were compared in the pretreatment and posttreatment groups. While the mean of serum total thiol in the pretreatment group was 500 ± 65 umol/L, it was 535 ± 45 umol/L in the posttreatment group. The higher total thiol level of the posttreatment group was found to be statistically significant (p = 0.008). While the mean TAS measurements in the pretreatment group was 1.57 ± 0.30 [mmol TEqv/L], it was 2.52 ± 0.29 [mmol TEqv/L] in the posttreatment group. The higher TAS level of the posttreatment group was found to be statistically significant (p = 0.001). While the mean PON measurements of the pretreatment group was 61.44 ± 27.62 [kU/L], it was 115 ± 46 [kU/L] in the posttreatment group. The higher PON level of the posttreatment group was found to be statistically significant (p = 0.001). While the mean serum native thiol in the pretreatment group was 386 ± 57 [umol/L], it was 545 ± 46 [umol/L] in the posttreatment group. The higher native thiol level of the posttreatment group was found to be statistically significant (p = 0.001). The native thiol/total thiol (SH/SH+SS) ratio was found to be significantly higher in the posttreatment group than in the pretreatment group (p = 0.001). While the mean TOS measurements of the pretreatment group was 4.08 ± 1.55 [μmol H202 Eqv/L], it was 2.76 ± 0.99 [μmol H202 Eqv/L] in the posttreatment group. The lower TOS level of the posttreatment group was found to be statistically significant (p = 0.001). While the mean OSI measurements of the pretreatment group was 2.71 ± 1.22 (AU), it was 1.10 ± 0.39 (AU) of the posttreatment group. The lower OSI level of the posttreatment group was found to be statistically significant (p = 0.001). The mean disulfide measurements in the pretreatment group were 25.39 ± 4.75 [umol/L], whereas in the posttreatment group, they were 10.17 ± 3.22 [umol/L]. The significantly lower disulfide level observed in the posttreatment group yielded a p-value of 0.001, indicating statistical significance. Additionally, both disulfide/native thiol (SS/SH) and disulfide/total thiol (SS/SH+SS) levels were notably lower in the posttreatment group compared to the pretreatment group, with a corresponding p-value of 0.001 (Table).

Furthermore, the study investigated the impact of age and length of the disease on oxidative stress measurements in the patients. A significant age-associated decline in native thiol levels among the cases was identified (r = 0.01, p < 0.05). Moreover, it was observed that age-related increases occurred in both disulfide (r = 0.331, p = 0.04) and disulfide/total thiol (r = 0.387, p = 0.017) levels within the cases. There was no statistically significant correlation detected between the length of the disease and the recorded measurements (p > 0.05). The effect of sexon oxidative stress measurements in the pre and posttreatment groups was examined. Overall, there was no statistically significant distinction observed between sexes in either of the groups (p > 0.05).

## 4. Discussion

Our study was conducted with 39 patients diagnosed with MS according to the revised McDonald criteria. In our study, we evaluated oxidative stress markers in patients with multiple sclerosis (MS) before and after six months of immunomodulatory treatment. Our findings have revealed significant changes in various oxidative stress parameters, including TAS, TOS, PON, and thiol levels. Also in our study, particularly after treatment, a statistically significant increase in TAS and total thiol levels, along with a significant decrease in TOS and OSI levels, was observed. Our results suggest that immunomodulatory therapies may reduce oxidative stress in MS patients, supporting their potential neuroprotective effects.

In many studies on the etiology of MS, factors such as genetic factors, oxidative stress, viral and bacterial infections, nutritional habits, chemical agents, organic solvents, vaccines and climatic conditions, in addition to immunopathogenesis, have been implicated and contradictory results have been obtained [12, 13]. Oxidative stress is a condition occurs as a result of the imbalance between reactive oxygen and nitrogen species, which are natural components of cellular processes, and the body’s antioxidant defense mechanism. Under normal physiological conditions, there is a balance between reactive oxygen and nitrogen species and the body’s antioxidant defense systems. Oxidative stress occurs when the balance is disrupted by increased quantities of reactive oxygen and nitrogen species or a deficiency in antioxidant mechanisms. As a result, the subsequent free radicals cause damage to important cellular components such as proteins, lipids, and nucleic acids, resulting in cell death via necrosis or apoptosis [8, 10]. In recent years, there has been a significant increase in research efforts dedicated to studying this field, with the specific goal of clarifying the exact impact of oxidative stress on the advancement of the disease.

In our study our findings align with previous studies that have explored oxidative stress in MS. In a study similar to our work, conducted by Jamroz-Wisniewska et al., 137 MS patients and 40 healthy controls were included, and no significant difference was found in serum PON activity between the patient and control groups. However, the study observed a decline in PON activity during relapse periods, correlating with increased oxidative stress. This finding emphasizes the role of oxidative stress in MS exacerbations and supports the concept that managing oxidative damage may contribute to disease stabilization [14]. Similarly, in our study, we found that PON activity increased significantly after treatment, which could be indicative of reduced oxidative stress following immunomodulatory therapy. These findings suggest that antioxidant defenses, including PON activity, may help control inflammatory processes during treatment and prevent further myelin loss and axonal damage. Further supporting this hypothesis, Ferretti et al. reported a marked decrease in PON activity in MS patients, particularly in comparison to controls, with a negative correlation observed between PON activity and EDSS (Expanded Disability Status Scale), suggesting that lower PON levels may be associated with greater disease severity [15]. Our study’s results are consistent with this, as we observed a significant improvement in PON activity posttreatment, which could be linked to reduced disease activity and oxidative stress. Interestingly, Martinez et al., who investigated PON genotypes in MS patients, found no significant differences in PON activity between MS patients and healthy controls, which contrasts with our findings of increased PON activity posttreatment. This discrepancy might stem from differences in study design, sample size, or the specific treatments administered. However, it remains clear that PON activity is influenced by the inflammatory state of the disease, as well as by therapeutic interventions [16]. In our study, the increase in PON activity following treatment likely reflects the reduction in the oxidative burden, possibly due to the control of inflammatory cascades and the prevention of further demyelination.

Moreover, Bożena Adamczyk et al. assessed oxidative stress parameters in patients with relapsing-remitting MS (RR-MS) undergoing various immunomodulatory treatments. Their study demonstrated that treatment with Natalizumab and Fingolimod led to lower levels of lipid hydroperoxides (LHP), malondialdehyde (MDA), and TOS compared to untreated patients, supporting the notion that immunomodulatory therapies can reduce oxidative stress [17]. These findings are consistent with ours, where we observed lower TOS and OSI levels after six months of treatment, further validating the protective effects of immunomodulatory therapies against oxidative damage. Similarly, our findings, the positive effects of immunomodulatory therapies on oxidative stress are further corroborated by the findings of Sadowska-Bartosz et al., who noted that interferon beta-1a and interferon beta-1b reduced serum oxidative stress parameters more significantly than mitoxantrone in secondary progressive MS patients [18]. We found a significant reduction in oxidative stress markers such as TOS and OSI, and an increase in antioxidant parameters like TAS and thiols, following treatment. These results emphasize the beneficial impact of immunomodulatory therapies in MS, not only in controlling disease activity but also in mitigating the harmful effects of oxidative stress.

In terms of thiol/disulfide balance, our study revealed a shift toward the antioxidative system after treatment, characterized by an increase in native thiol levels and a decrease in disulfide levels. This shift is consistent with the findings of Haider et al., who suggested that oxidative stress leads to an imbalance in thiol/disulfide equilibrium, contributing to demyelination and neuronal damage in MS [19]. By favoring the antioxidative system, immunomodulatory treatments may help restore this balance, thereby mitigating oxidative damage and protecting cellular components from further harm. Thiols, including glutathione, play a crucial role in maintaining redox homeostasis in the brain, and their role in neuroprotection has been widely documented in neurological diseases.

Our findings are consistent with the study by Aldabah et al. The results of this study, which involved 14 patients with relapsing-remitting multiple sclerosis treated with interferon beta (IFN-β) for 6 months, demonstrate a reduction in erythrocyte vitamin E levels prior to treatment, with a subsequent return to levels comparable to the control group after 6 months of therapy. This finding suggests that IFN-β treatment may exert a sparing effect on erythrocyte vitamin E levels, which could be linked to the antioxidative properties of the therapy. Additionally, the study reported significantly higher Expanded Disability Status Scale scores in newly diagnosed patients compared to those on IFN-β therapy, highlighting the beneficial effect of IFN-β in slowing disease progression and reducing disability [20]. Our results demonstrate significant improvements in several oxidative stress markers after treatment, including total thiol, total antioxidant status (TAS), and paraoxonase (PON) levels, all of which were higher in the posttreatment group. These findings suggest that immunomodulatory therapy, including IFN-β, may not only exert a direct effect on reducing disease activity and progression, as indicated by improved EDSS scores but also enhance the antioxidant defense system, which could mitigate oxidative stress in RRMS patients.

Finally, our study has some limitations. It was conducted at a single center with a relatively small sample size. Additionally, the study focused on serum oxidative stress markers, which may not fully capture the oxidative equilibrium within the brain. Future studies, particularly those involving larger cohorts and assessments of oxidative stress markers in cerebrospinal fluid (CSF) or at the receptor level, are necessary to further elucidate the precise role of oxidative stress in MS and the effects of various treatments on oxidative balance.

In conclusion, our study supports the hypothesis that immunomodulatory treatments have a beneficial impact on oxidative stress parameters in MS patients. Specifically, these therapies appear to reduce oxidative damage by improving the balance of antioxidant and oxidant systems, as evidenced by the changes in TAS, TOS, PON, and thiol levels. These findings contribute to the growing body of evidence suggesting that reducing oxidative stress may be an important therapeutic strategy in managing MS and potentially slowing disease progression.

## Conclusion

In conclusion, the present study has shown us that immunomodulatory treatments used in MS have positive effects on the oxidative system. Moreover, TAS, TOS, OSI, and PON activity, which are indicators of oxidative stress, might serve as potential markers for assessing therapy response and monitoring the clinical progression of patients with MS in the future.

## Figures and Tables

**Table:** Comparison of oxidative stress measurements before and after treatment. TAS, TOS, OSI and PON values in groups.

	Patient		Comparison results
	Before treatment	After treatment	Before treatment-after treatment
	Mean ± SD	Mean ± SD	P.
**Antioxidative system**			
Total thiol [umol/L]	500 ± 65	535 ± 45	0.008
STONE [mmol TEqv/L]	1.57 ± 0.30	2.52 ± 0.29	0.001
PON [kU/L]	61.44 ± 27.62	115 ± 46	0.001
Native thiol [umol/L]	386 ± 57	545 ± 46	0.001
Native/total thiol (%)	0.785 ± 0.141	1.024 ± 0.112	0.001
**Oxidative system**			
TOS [μmol H2O2 Eqv/L]	4.08 ± 1.55	2.76 ± 0.99	0.001
OSI (AU)	2.71 ± 1.22	1.10 ± 0.39	0.001
Disulphide [umol/L]	25.39 ± 4.75	10.17 ± 3.22	0.001
Disulfide/native thiol (%)	0.068 ± 0.018	0.019 ± 0.006	0.001
Disulfide/total thiol (%)	0.052 ± 0.014	0.019 ± 0.006	0.001
